# Diagnosis Identity Perception in Adolescents with ADHD and Its Relationship to Executive Functions, Self-Management, and Quality of Life

**DOI:** 10.3390/children12111532

**Published:** 2025-11-13

**Authors:** Yael Zabar-Cahanovich, Adi Stern, Liron Lamash

**Affiliations:** 1 Department of Occupational Therapy, Faculty of Social Welfare & Health Sciences, University of Haifa, Haifa 3498838, Israel; yaelcahanovich@gmail.com; 2 Department of Occupational Therapy, Faculty of Health Sciences, Ben-Gurion University of the Negev, Beer Sheva 8410501, Israel; adister@bgu.ac.il

**Keywords:** diagnosis identity, attention-deficit/hyperactivity disorder, adolescence, executive function, self-management, quality of life

## Abstract

**Highlights:**

**What are the main findings?**

• Adolescents with ADHD reported significantly higher identity Acceptance scores compared to Rejection, Engulfment, and Enrichment, *F* (2.38, 149.89) = 32.41, *p* < 0.001.
• The total diagnosis identity score was strongly associated with self-management (r = 0.61, *p* < 0.001).
• The Engulfment dimension showed strong and consistent associations with both executive dysfunction and lower quality of life, despite the absence of correlations at the total score level.
• Regression analyses indicated that self-monitoring, social QoL, and self-evaluation together explained 45% of the variance in ADHD diagnosis identity. The Engulfment dimension of identity was a significant negative predictor of executive functioning (*R*^2^ = 0.15), self-management (*R*^2^ = 0.35), and QoL (*R*^2^ = 0.17).

**What are the implications of the main findings?**

• The findings underscore the complex relationship between diagnosis identity and emotional as well as functional outcomes in adolescents with ADHD.
• Negative perceptions of one’s diagnosis identity predict poorer executive functioning, self-management, and QoL, while core aspects of self-management and social well-being also significantly shape how adolescents with ADHD perceive their diagnosis.
• The results highlight the need for holistic, individualized interventions that address both the development of positive diagnosis identity and the strengthening of executive and self-management skills of adolescents with ADHD.
• Promoting a positive diagnosis identity may improve functional and emotional outcomes, which in turn may reinforce a more adaptive and accepting view of the diagnosis itself.

**Abstract:**

**Background/Objectives**: This study aimed to describe how adolescents with attention-deficit/hyperactivity disorder (ADHD) perceive their diagnosis identity and examine its correlation with executive functions (EFs), self-management abilities, and quality of life (QoL). Methods: A total of 66 adolescents with ADHD, aged 12 to 18 years (*M* = 15.21, *SD* = 1.84), completed self-report questionnaires, including the Illness Identity Questionnaire, Behavior Rating Inventory of Executive Function, Self-Control and Self-Management Scale, and Pediatric QoL Inventory. We used ANOVA with Bonferroni post hoc tests to assess differences in diagnosis identity domains and Pearson correlations to examine correlations between diagnosis identity, EFs, self-management, and QoL. **Results**: Adolescents reported significantly higher ADHD *Acceptance* feelings compared to *Rejection*, *Engulfment*, and *Enrichment*, *F* (2.38, 149.89) = 32.41, *p* < 0.001. Total diagnosis identity score was strongly associated with self-management (*r* = 0.61, *p* < 0.001). While no significant correlations were found with overall EF or QoL, significant associations did emerge with their sub-scores. Regression analyses indicated that self-monitoring, social QoL, and self-evaluation together explained 45% of the variance in diagnosis identity. The *Engulfment* dimension of identity was a significant negative predictor of executive functioning (*R*^2^ = 0.15), self-management (*R*^2^ = 0.35), and QoL (*R*^2^ = 0.17). **Conclusions**: Promoting a positive diagnosis identity may improve functional and emotional outcomes in adolescents with ADHD. In turn, better functional and emotional outcomes may help them embrace a diagnosis identity that is more positive.

## 1. Introduction

The neurodevelopmental condition, attention-deficit/hyperactivity disorder (ADHD), is characterized by difficulties with sustaining attention and/or hyperactivity-impulsivity that interfere with functioning and development [1]. As one of the most common neurodevelopmental disorders, the prevalence of ADHD among children and adolescents is estimated to be around 8% globally [2]. Although ADHD was traditionally considered a childhood disorder, it is currently recognized as a lifelong condition [3,4].

The World Health Organization [5] defines adolescence as the transitional stage between childhood and adulthood, occurring between the ages of 10 and 19. This period is marked by significant growth and development across all areas of life. In addition to physical changes, adolescents undergo substantial cognitive, emotional, and social transformations. Additionally, it is a time characterized by identity formation, development of social roles and interactions, self-exploration, and cultivation of autonomy and self-reliance [6].

For adolescents with ADHD, this stage can present particularly profound challenges. Increased academic and social demands make this period especially vulnerable because adolescents with ADHD often face higher rates of social rejection, isolation, and educational difficulties [7,8]. These challenges are compounded by low self-esteem, reduced motivation to achieve, social-skills deficits, and social withdrawal [9]. Studies also indicate that these deficits in social, physical, and emotional domains negatively affect quality of life (QoL), particularly during adolescence. Adolescents with ADHD often have a significantly lower QoL compared to their peers without an ADHD diagnosis [6,10].

One central deficit commonly associated with ADHD is impairments in executive functions (EFs) [11,12]. Approximately 67% of adolescents with ADHD exhibit clinically significant EF deficits, particularly in planning, organization, and working memory domains [13]. EFs are high-level cognitive processes involving complex, goal-directed behaviors such as planning, control, problem-solving, working memory, and inhibition which are necessary to perform tasks in diverse situations and environments [14]. They enable individuals to make informed decisions and use strategies to enhance performance, learning, and daily functioning [14]. Thus, EF deficits can have long-term implications for daily life and significantly affect planning, organization, decision-making, academic achievement, and social skills, potentially leading to maladaptive behaviors [15,16].

Adolescence is a period of significant EFs development [17]. Adolescents with ADHD often exhibit more deficits in tasks requiring EFs than their typically developing peers [18,19,20]. They frequently struggle with maintaining attention, inhibiting responses, and sustaining perseverance [11,18,19,20]. These difficulties may hinder them in developing various aspects of self-perception and identity, which are critical developmental tasks during this stage of life. Additionally, the challenges associated with EF deficits may impair daily functioning, academic performance, self-regulation, and self-management [16].

Self-management is a lifelong task that requires EFs, such as problem-solving and decision-making [21,22]. It involves managing time and adapting as circumstances change, with an acute awareness of experiences and their impact on one’s physical and mental state [23]. These skills are designed to improve EFs, reduce core symptoms, and enhance QoL and self-identity perception. For youth with chronic health conditions, self-management skill development is a long, complex process shaped by psychological factors, social networks, and health system involvement [24,25,26,27,28]. Ultimately, this development can lead to positive changes in health and social outcomes [19].

*Identity* refers to internal self-awareness, encompassing an individual’s direction in life and future goals [29]. Self-identity is a composite of skills, knowledge, and beliefs shaped by personal traits, characteristics, life roles, physical appearance, values, goals, past experiences, and ongoing interactions with significant others [29,30]. It enables individuals to work toward achieving their goals while recognizing their strengths and limitations and fosters a sense of capability and efficacy. This recognition is essential for self-definition and functioning as a successful societal adult. Adolescents with chronic illnesses tend to integrate their diagnosis into their identity [31], which may include their knowledge and goals related to their condition, guiding their actions, values, and behaviors in various situations [30]. Forming a well-integrated and consolidated identity by, for example, committing to plans and actively participating in life decisions can protect young people living with chronic illnesses against distress and depressive symptoms [32].

The term *illness identity* has recently been used to describe how a person integrates a chronic illness into their identity [33]. It refers to the attitudes and roles a person develops about their self, their health condition, and their integration of the condition into their daily life [34,35]. Using this concept, Oris et al. [34] described the degree to which a person integrates their chronic illness with their sense of self. They described four dimensions of illness identity: (a) *Acceptance*, how much a person accepts their illness and its challenges as part of their identity without being overwhelmed by it; (b) *Enrichment*, how much the person feels the illness allowed them to develop and discern advantages for their identity in it; (c) *Rejection*, how much the person rejects the illness and does not perceive it as part of their identity; and (d) *Engulfment*, how much the person’s illness engulfs their identity and permeates all areas of their life. Whereas *Acceptance* and *Enrichment* are considered positive dimensions of identity perception, *Rejection* and *Engulfment* are considered negative [36].

Several studies have examined illness identity perception among adolescents and adults with chronic health conditions, such as type 1 diabetes, epilepsy, and celiac disease, and how these illnesses integrate into their sense of self [34,36,37,38]. These studies demonstrated that illness identity plays a central role in adapting to chronic illnesses. Adolescents who felt *Engulfment* were more likely to experience symptoms of depression and anxiety. On the other hand, *Acceptance* of the diagnosis was linked to better social abilities, improved coping skills, and increased social participation. Other studies have explored illness identity (herein renamed *diagnosis identity*) within neurodevelopmental conditions such as autism spectrum disorder and ADHD. A positive diagnosis identity in autistic individuals was associated with greater psychological well-being, higher QoL, and broader social participation [39,40,41]. Similarly, recent studies have examined diagnosis identity and its psychosocial implications among adolescents with ADHD, emphasizing how the interpretation of one’s diagnosis shapes self-concept, social relationships, and adjustment [42,43,44]. These findings indicate that illness identity is not limited to the experience of chronic health conditions but reflects broader identity processes common to neurodevelopmental conditions.

In sum, adolescence is a critical period for development, which may be more complex for individuals with ADHD. Adolescents with ADHD often face various emotional and functional impairments, which further contribute to low self-esteem and QoL. Hence, developing a positive self-image, self-esteem, and identity perception can be particularly challenging for them [45]. The self-identity of adolescents with ADHD often incorporates aspects related to their diagnosis, challenges, and goals, influencing their behavior in various contexts. The absence of a stable identity may lead to difficulties in EFs, impair self-management abilities, and negatively impact QoL. These emotional and functional deficits may, in turn, further hinder identity formation. Despite the importance of this issue, it has yet to be thoroughly explored. Therefore, the aims of this study were to examine how adolescents with ADHD perceive their diagnosis identity and how it relates to their EFs, self-management abilities, and QoL.

## 2. Materials and Methods

### 2.1. Study Design

This was a cross-sectional study using an anonymous online survey. The sample was recruited using convenience sampling, and eligibility was confirmed through a brief parental screening process before data collection.

### 2.2. Participants

A sample size of approximately 70 participants was determined using the G-Power test based on a one-tailed correlation with an effect size of 0.03, alpha level of 0.05, and power of 80%. The final sample included 66 adolescents aged 12 to 18 years (*M* = 15.21 years, *SD* = 1.84) with a formal diagnosis of ADHD. Of these, 50 (75.8%) participants were male, 15 (22.7%) were female, and one (1.5%) did not want to specify gender. Other comorbid neurodevelopmental or chronic health conditions (e.g., autism spectrum disorder, intellectual disability, or chronic medical illnesses) served as exclusion criteria to minimize the influence of additional conditions on the experience related to having ADHD. Due to the anonymous nature of the online survey, verification of ADHD diagnosis provided by a qualified authority, and the absence of comorbidities, were confirmed at the parental screening stage. Table 1 presents additional participant demographic characteristics.

### 2.3. Measures

#### 2.3.1. Demographic Questionnaire

Items in the demographic questionnaire developed for this study included parental confirmation of the child’s ADHD and additional chronic or neurodevelopmental diagnoses. Other items collected general sociodemographic data, such as gender, age, and education settings.

#### 2.3.2. Illness Identity Questionnaire

The Illness Identity Questionnaire (IIQ) [34] is a self-report questionnaire designed to measure the extent to which a chronic health or developmental condition integrates into the respondent’s daily life and identity. For this study, the IIQ was explicitly adapted for ADHD following the authors’ guidelines and with their permission. The questionnaire consists of 25 statements rated on a 5-point Likert scale from 1 (strongly disagree) to 5 (strongly agree). The IIQ assesses four dimensions of diagnosis identity: (a) *Rejection*, including five items; (b) *Engulfment*, including eight items; (c) *Acceptance*, including five items; and (d) *Enrichment*, including seven items. Each dimension’s score is calculated as the average of its related items; a higher score indicates a greater expression of that dimension. Items from the negative dimensions (*Rejection* and *Engulfment*) are reverse-scored; mean scores are calculated across all items to achieve an overall score; higher overall scores reflect more positive identity perception. The IIQ has demonstrated high discriminant validity and internal consistency; reliability coefficients ranged from 0.75 to 0.95 among adolescents with chronic conditions [34,46]. In the current study, internal reliability was good for all IIQ items (α = 0.81) and adequate for each component (α = 0.71 − 0.84).

#### 2.3.3. Behavior Rating Inventory of Executive Function-Self-Report

The Behavior Rating Inventory of Executive Function (BRIEF-SR) [47] is a self-report questionnaire designed to assess EFs in adolescents aged 11 to 18 years. The questionnaire comprises 80 statements in which participants are asked to rate the frequency of specific behaviors on a 3-point scale: 1 (never), 2 (sometimes), and 3 (often or always). The items are divided into eight categories that assess different aspects of executive functioning. Three categories, inhibition, shifting, and emotional regulation, form the behavioral regulation index (BRI); five categories, initiation, working memory, planning/organization, environmental organization, and monitoring, form the metacognition index (MI). Raw scores are computed for each category by summing the item ratings. Additionally, composite scores are calculated for the BRI, MI, and the global executive composite (GEC), which reflects overall executive functioning. Using gender- and age-specific norms to convert these raw scores provides a standardized score for executive functioning; a lower score reflects better executive functioning. Scores below 60 represent normal functioning, scores from 60 to 65 indicate at-risk functioning, and scores greater than 65 signify impaired functioning. The BRIEF-SR has demonstrated strong content validity, construct validity, and high internal reliability across its scales, with reported reliabilities of Cronbach’s alphas of 0.93 (BRI), 0.94 (MI), and 0.96 (GEC) [47]. In the current study, internal reliability was similarly high, with α = 0.96 for all items and α = 0.95 to 0.96 across the categories.

#### 2.3.4. Self-Control and Self-Management Scale

The Self-Control and Self-Management Scale (SCMS) [48] is a self-report questionnaire to assess self-management abilities in daily life. The questionnaire consists of 16 statements rated on a scale from 0 (does not describe me at all) to 5 (describes me very well). These items are divided into three subscales: self-monitoring (six statements), self-evaluating (five statements), and self-reinforcing (five statements) scales. Together, they produce an overall score reflecting self-management skills. Scores are calculated by summing the ratings of all relevant items; higher scores indicate better self-management abilities [49]. The SCMS has demonstrated construct and discriminant validity, along with high internal reliability for all items (α = 0.85) and adequate to good reliability for the subscales (α = 0.72 − 0.80). The current study found reliability acceptable, with a Cronbach’s alpha of 0.75 for all items and subscale reliability ranging from 0.59 to 0.87.

#### 2.3.5. Pediatric Quality of Life Inventory

The self-reported Pediatric Quality of Life Inventory (PedsQL), designed for adolescents aged 13 to 18 years [50], assesses health-related QoL. It applies to healthy adolescents and those with chronic health conditions. The questionnaire includes 23 items that evaluate four dimensions of QoL: eight physical-functioning items, five emotional-functioning items, five social-functioning items, and five school-functioning items. Adolescents are asked to indicate the extent to which each statement represents a difficulty or problem on a scale from 0 (never a problem) to 4 (almost always a problem). These ratings are converted to a linear score ranging from 0 to 100 (0 = 100, 1 = 75, 2 = 50, 3 = 25, 4 = 0), with higher scores indicating better QoL. In addition to an overall score, mean scores for the four dimensions are calculated. The PedsQL has demonstrated high internal reliability for self-reports (α = 0.88) and possesses strong construct and discriminant validity between groups with varying morbidity levels [50]. In the current study, the PedsQL demonstrated acceptable internal reliability for all items (α = 0.72) and good reliability across the categories (α = 0.79 − 0.84).

### 2.4. Procedure

The University of Haifa Institutional Ethics Committee approved this study (No. 274/19-1). We recruited participants by directing advertisements to parents of adolescents with ADHD via relevant interest groups, forums, and social media. The advertisements included a link to an explanatory letter about the study and several screening questions designed to confirm eligibility. Parents who expressed interest in their child’s participation were asked to complete an informed consent form. Subsequently, they forwarded the survey link to their adolescents, who independently completed the questionnaire on Google Forms. Because the online form required a response for every item, no forms were submitted with missing data. Completion time was approximately 25 min. Responses were transmitted automatically and anonymously to a secure database only the research team could access. Data collection took place between February and April 2024.

### 2.5. Data Analyses

We analyzed the data using IBM SPSS (Version 27). Descriptive statistics summarized the sample characteristics, including the variables’ ranges, means, and standard deviations. An analysis of variance with Bonferroni post hoc analysis for repeated measures was performed to assess differences in diagnosis identity perception measures. Additionally, we conducted Pearson correlation analyses to explore the correlation between dimensions of diagnosis identity perception (IIQ) and EFs (BRIEF-SR), self-management (SCMS), and QoL (PedsQL).

## 3. Results

### 3.1. Diagnosis Identity Perception of Adolescents with ADHD

A repeated measures ANOVA was conducted to examine differences between the four dimensions of diagnosis identity (*Rejection, Acceptance, Engulfment,* and *Enrichment*), as measured by the IIQ. Mauchly’s test indicated a violation of the sphericity assumption, χ^2^(5) = 22.67, *p* < 0.001; therefore, we applied the Greenhouse–Geisser correction (ε = 0.795). The results showed a statistically significant effect of identity dimension on IIQ scores, *F* (2.38, 149.89) = 32.41, *p* < 0.001, partial η^2^ = 0.607, indicating a large effect size. Multivariate tests (Wilks’ Lambda = 0.393, *p* < 0.001) also confirmed a significant overall difference across the four dimensions. Post hoc comparisons using Bonferroni-adjusted pairwise tests showed that *Acceptance* scores (*M* = 3.74, *SD* = 0.88) were significantly higher than *Rejection* (*M* = 2.54, *SD* = 0.91), *Engulfment* (*M* = 2.38, *SD* = 0.81), and *Enrichment* (*M* = 2.75, *SD* = 0.93) scores (*p* < 0.001 for all comparisons). *Engulfment* scores were significantly lower than all other dimensions (*p* < 0.001), while *Enrichment* did not significantly differ from *Rejection* (*p* > 0.05). No significant gender differences were observed in IIQ scores, nor were there significant differences between adolescents who reported taking ADHD medication and those who did not.

#### 3.1.1. Relationship Between Diagnosis Identity Perception and EFs, Self-Management, and QoL

We conducted correlational analyses to explore the relationships among adolescents’ diagnosis identity perceptions (as measured by the IIQ) and their reported EFs, self-management, and QoL.

#### 3.1.2. Relationship Between Diagnosis Identity and EFs

No significant correlations were found between the IIQ total score and the BRIEF-SR total (GEC), behavioral regulation (BRI), or metacognitive abilities (MI) scores. Further analysis explored the correlation between the IIQ dimensions and the specific BRIEF-SR scales. Most of the significant correlations were associated with the *Engulfment* dimension, suggesting that the participants who felt their ADHD diagnosis was more central or overwhelming tend to experience greater executive dysfunctions, and conversely, greater difficulties in executive functioning may reinforce a more engulfed or consuming sense of diagnosis identity. Table 2 presents these results, including detailed correlations between the IIQ dimensions and the BRIEF-SR scales.

#### 3.1.3. Relationship Between Diagnosis Identity and Self-Management

A strong positive correlation was found between the IIQ total score and the SCMS total score (*r* = 0.61, *p* < 0.001), suggesting that adolescents with a more integrated diagnosis identity tend to report better self-regulation. Likewise, adolescents with stronger self-regulation tend to perceive their diagnosis identity more positively. Among the IIQ dimensions, *Acceptance* and *Enrichment* were most positively associated with SCMS subscales, while *Rejection* and *Engulfment* showed negative correlations. Detailed correlations between IIQ dimensions and SCMS subscales are presented in Table 2.

#### 3.1.4. Relationship Between Diagnosis Identity and QoL

No significant correlation was found between the IIQ total score and overall PedsQL scores, suggesting that general diagnosis identity integration is not directly linked to perceived QoL. However, we observed significant negative correlations for the *Engulfment* dimension with the physical (*r* = −0.42, *p* < 0.01), emotional (*r* = −0.31, *p* < 0.05), and social (*r* = −0.28, *p* = 0.05) domains of QoL. These findings suggest that adolescents who feel more engulfed by their ADHD diagnosis tend to report lower well-being, and those experiencing reduced QoL may be more likely to perceive their diagnosis as overwhelming. Table 2 presents the full correlation results.

### 3.2. Diagnosis Identity Perception: Bidirectional Prediction

#### 3.2.1. Predicting Diagnosis Identity by EFs, Self-Management, and QoL

A stepwise multiple regression analysis was conducted to examine predictors of diagnosis identity perception (IIQ total score). Three variables were entered sequentially as significant predictors: self-monitoring (SCMS), social QoL (PedsQL), and self-evaluation (SCMS). The final model accounted for 45.3% of the variance in diagnosis identity perception. No evidence of multicollinearity was observed, as all VIF values were close to 1. Table 3 presents a summary of the regression models.

#### 3.2.2. Predicting EFs, Self-Management, and QoL by Diagnosis Identity Dimensions

A series of stepwise multiple regression analyses was conducted to examine the predictive value of diagnosis identity perception dimensions (IIQ) for executive functioning, self-monitoring, and QoL. *Engulfment* emerged as a significant negative predictor of both executive functioning and QoL; specifically, adolescents who reported greater identification with their diagnosis showed poorer cognitive and well-being outcomes. In contrast, *Enrichment* was a positive predictor of self-management skills, reflecting that perceiving one’s diagnosis as meaningful and growth-promoting was associated with better self-regulatory abilities. Together, these models explained approximately 15% to 35% of the variance across the outcome measures. No evidence of multicollinearity was observed in any model (all VIFs < 1.05). The results are summarized in Table 4.

## 4. Discussion

The present study reveals that adolescents with ADHD are more likely to perceive their diagnosis identity in positive terms, with *Acceptance* emerging as the most prominent dimension compared to *Rejection* and *Engulfment*. This tendency aligns with prior research on other neurodevelopmental and chronic conditions, such as celiac disease [38] and autism [51], where a positive component of diagnosis identity has also been observed. Moreover, our results complement prior work with adolescents with ADHD, who often characterize their experience in terms of openness, honesty, empathy, high energy and motivation, creativity, agreeableness, hyperfocus, and strong willingness to help others [52]. Recognizing and validating these strengths can foster greater acceptance, more adaptive coping strategies, and, in some cases, even a sense of pride in one’s diagnosis [53]. The positive aspects of ADHD have also been emphasized in the growing influence of the Neurodiversity Movement [54], which highlights individuals’ strengths, talents, and unique contributions and views neurological differences as natural and valuable expressions of human variation [55].

However, our findings also reveal that the diagnosis identity perceptions of adolescents with ADHD are not exclusively positive or negative. Instead, most participants demonstrated a nuanced and multidimensional profile, experiencing both *Acceptance* and *Enrichment* alongside feelings of *Rejection* and *Engulfment*. This internal complexity echoes recent qualitative research showing that adolescents with ADHD often navigate a dynamic mix of pride, self-acceptance, and meaning, while simultaneously contending with stigma, self-doubt, or a sense of difference [44,56,57]. Such findings highlight that diagnosis identity in ADHD is shaped by ongoing negotiation between positive self-concepts and the psychological or social challenges associated with the diagnosis.

The present study found that feelings of *Engulfment*, meaning the sense that ADHD becomes central and overwhelming to one’s identity, were significantly associated with EF deficits among adolescents. This finding supports the conceptualization of ADHD diagnosis identity as closely intertwined with self-regulation processes. Qualitative and quantitative research has suggested that when adolescents experience their diagnosis as engulfing, they may also struggle more with inhibitory control and emotional regulation, core components of EF [13,56,58]. Our results further indicate that better inhibition and emotional regulation are linked to more positive perceptions of the ADHD diagnosis, echoing previous studies emphasizing the centrality of these EFs for developing a coherent and adaptive self-concept [58,59,60]. Together, these findings highlight the value of considering both the cognitive and diagnosis identity dimensions of ADHD in intervention planning. Specifically, addressing feelings of *Engulfment* and fostering executive functioning skills, particularly inhibition and emotional regulation, may help adolescents with ADHD develop a more positive and balanced identity.

Our study demonstrates that adolescents with ADHD who report higher levels of *Acceptance* and *Enrichment* also tend to exhibit better self-management abilities, particularly in self-monitoring and self-reinforcement. Conversely, greater feelings of *Rejection* and *Engulfment* are linked to poorer self-management. These findings align with previous research in other chronic conditions, where a positive illness or diagnosis identity has been associated with improved self-management and a more adaptive self-concept [61,62,63]. Self-monitoring, a key aspect highlighted in the current study, is crucial to behavior change and self-regulation among youth with ADHD [64,65]. Recent qualitative research further supports that when adolescents constructively internalize their diagnosis, they develop a stronger sense of agency and are better able to adopt effective coping strategies [44]. Additionally, the positive association between diagnosis identity and self-reinforcement underscores the importance of fostering not only knowledge and skills but also a healthy, empowered self-perception as part of interventions for adolescents with ADHD. Given the limited research on self-reinforcement in neurodevelopmental populations, future studies should investigate how enhancing positive diagnosis identity can strengthen core self-management processes and contribute to better outcomes for this group.

A growing body of research on chronic and neurodevelopmental conditions demonstrates that negative diagnosis identity, particularly the sense of *Engulfment,* can have substantial adverse effects on QoL, including greater psychosocial distress and lower functioning in physical, social, and emotional domains [34,37,66]. The current study extends these findings to adolescents with ADHD, revealing that negative dimensions of diagnosis identity are strongly linked to reduced physical, social, and emotional QoL. Importantly, these associations emerged even though positive perceptions were more prevalent overall among participants. Similarly to reports in other conditions (e.g., [67]), adolescents with ADHD who exhibit higher acceptance of their diagnosis tend to report better QoL. Conversely, those experiencing greater *Engulfment* and *Rejection* face a heightened risk for poor psychosocial outcomes. These results highlight the importance of developing targeted interventions that not only cultivate strengths and talents but also address and reduce negative diagnosis identity, as these negative emotions are closely related to adolescents’ daily functioning and well-being [34,37,51].

The decision to also examine bidirectional predictive associations between ADHD diagnosis identity and functional outcomes reflects the view that these domains influence one another dynamically. A coherent and positively integrated sense of diagnosis identity can enhance self-awareness, motivation, and coping, contributing to improved executive functioning and overall well-being (e.g., [44]). At the same time, well-developed executive abilities and psychosocial adjustment provide the cognitive and emotional resources necessary to reflect on one’s diagnosis and develop a stable, adaptive identity (e.g., [68]). The results of the regression analyses highlight this bidirectional relationship between diagnosis identity perception and functional and emotional outcomes in adolescents with ADHD. On one hand, dimensions of diagnosis identity, particularly *Engulfment* and *Enrichment*, were significant predictors of executive functioning, self-management, and QoL. A more positive diagnosis identity predicted better daily functioning, while a negative diagnosis identity predicted greater impairment. On the other hand, self-monitoring and self-evaluation, as well as social QoL, predicted overall diagnosis identity perception, jointly accounting for nearly half the variance. These findings indicate that adolescents’ day-to-day skills and well-being not only reflect their sense of diagnosis identity but also actively shape it. This relationship aligns with recent theoretical frameworks suggesting that identity development in chronic and neurodevelopmental conditions is a dynamic process, continually influenced by both internal capacities and external experiences [34,37,44].

Practically, these findings highlight the importance of holistic and differentiated interventions across clinical, educational, and family contexts. For clinicians and educators, the results emphasize the need to assess adolescents with ADHD in terms of not only their clinical symptoms and functional abilities but also their diagnosis identity—how they perceive and internalize their diagnosis. Therefore, interventions should aim to reduce negative meanings associated with ADHD, foster interpretations that are more positive and empowering, and strengthen self-efficacy. Such approaches can enhance daily functioning, social experiences, and overall QoL. Promoting executive and self-management skills may further support adaptive development of diagnosis identity. For families, addressing adolescents’ diagnosis identities can guide parents and caregivers in providing emotional and practical support that reinforces self-regulation, confidence, and inclusion. Collaboration among teachers, counselors, and family members around this shared understanding may foster a more adaptive and positive identity formation process, ultimately improving the well-being and daily functioning of adolescents with ADHD. It is important for future research to explore the mechanisms underlying these correlations, including the role of environmental factors (e.g., family support, school climate) and the potential for targeted interventions to disrupt maladaptive feedback loops between diagnosis identity perception and daily functioning.

This study’s limitations should be acknowledged. The ADHD diagnoses were based solely on parental confirmation of a formal clinical diagnosis, without independent verification. The relatively small sample size (*N* = 66) and use of convenience sampling may have reduced the statistical power to detect subtle effects and limited the generalizability of the findings to the broader population of adolescents with ADHD. Participants with parent-reported neurodevelopmental or chronic comorbidities were excluded to focus specifically on illness identity related to ADHD. This deliberate decision enhanced conceptual clarity but limited the generalizability of the findings to adolescents with ADHD who present additional conditions. Moreover, participants who voluntarily chose to participate might differ in motivation, awareness, or coping strategies from those who did not, potentially introducing selection bias. The requirement for adolescents to independently complete a relatively long online questionnaire (approximately 25 min) may have further excluded those with more severe attentional or behavioral difficulties. Although symptom severity was assessed, it was not significantly related to diagnosis identity or outcome measures. Therefore, we did not include it in the main analyses. Additionally, the study did not distinguish between ADHD subtypes (inattentive, hyperactive-impulsive, or combined). These subtypes may differ in behavioral and emotional characteristics and could potentially mediate or moderate the relationships observed in this study.

The gender distribution (76% male) is higher than typically reported in epidemiological studies (e.g., [69]) but consistent with clinical samples where boys are typically overrepresented; this may limit generalizability, particularly to girls with ADHD who are often underdiagnosed [70]. While no gender differences were observed in diagnosis identity (IIQ), some independent variables (e.g., self-regulation, executive functioning) did show differences, suggesting gender may act as a confounding or moderating factor. Similarly, although stimulant medication use was not associated with any of the study variables, the modest sample size limited examination of dosage or duration effects, and thus, medication influences cannot be entirely ruled out. Additional limitations include the study’s use of self-report measures, which could introduce response bias, and its cross-sectional design, which precludes causal inference regarding the directionality of relationships.

Future studies should recruit larger and more diverse samples through randomized or stratified procedures across multiple settings to enhance external validity and enable subgroup analyses (e.g., gender or medication status). They should also consider ADHD subtype distinctions to better understand their possible influence on the study variables. Incorporating longitudinal or experimental designs and developing parallel tools for other diagnosis groups to enable cross-condition comparisons. Finally, qualitative approaches could further enrich understanding of how adolescents with ADHD experience and integrate their diagnoses into daily life.

## 5. Conclusions

This study’s findings underscore the complex relationship between diagnosis identity and emotional and functional outcomes in adolescents with ADHD. Not only do negative perceptions of diagnosis identity, such as *Engulfment* and *Rejection*, predict poorer executive functioning, self-management, and QoL, but core aspects of self-management and social well-being also significantly shape how adolescents perceive their diagnosis. These results spotlight the importance of and need for holistic, individualized interventions that address both the development of positive diagnosis identity and the strengthening of executive and self-management skills. Clinicians, educators, and families should be aware of the ongoing interplay between diagnosis identity and daily functioning, supporting adolescents in embracing their diagnosis and building practical coping strategies to improve QoL.

## Figures and Tables

**Table 1:** Participants’ demographic characteristics.

Characteristic	Range	*M* (*SD*)
Age of diagnosis (years)	4–17	10.11 (3.27)
Age of exposure to diagnosis (years)	3–17	10.20 (3.06)
	*n* (%)	
Educational setting		
*Public school*	62 (93.9)	
*Anthroposophical/democratic*	3 (4.5)	
*External school/home school*	1 (1.5)	
Class type		
*Mainstream class/full matriculation*	58 (87.9)	
*Special education*	3 (4.5)	
*Gifted/sport*	5 (7.6)	
Stimulant medication		
*Yes*	24 (36.4)	
*No*	42 (63.6)	

**Table 2:** Correlations between the IIQ and the BRIEF-SR, SCMS, and PedsQL.

	IIQ				
	*Rejection*	*Acceptance*	*Engulfment*	*Enrichment*	*Total IIQ*
**BRIEF-SR**					
Inhibition	−0.25 *	0.20	0.38 **	0.26 *	0.11
Shift	−0.15	0.10	0.42 **	0.20	0.00
Emotional control	−0.16	0.13	0.32 **	0.26 *	0.08
***BRI Score***	−0.21	0.21	0.42 ***	0.24	0.05
Organization of material	−0.10	0.07	0.27 *	0.03	−0.04
Task completion	0.05	−0.05	0.37 **	−0.03	−0.19
Working memory	−0.12	0.13	0.40 ***	0.16	0.00
Plan/organize	−0.01	0.00	0.38 **	0.04	−0.18
Monitor	−0.14	−0.03	0.26 *	−0.03	−0.10
***MI Score***	0.05	0.13	0.43 ***	0.09	−0.12
***GEC Score***	−0.13	0.11	0.46 ***	0.17	−0.04
**SCMS**					
Self-monitoring	−0.30 *	0.47 ***	−0.29 *	0.46 **	0.58 **
Self-evaluation	−0.17	0.14	−0.02	0.23	0.20
Self-reinforcing	−0.22	0.28 *	−0.11	0.31 **	0.36 **
***SCMS Total***	−0.37 **	0.45 ***	−0.35 **	0.39 ***	0.61 ***
**PedsQL**					
Physical	−0.02	0.20	−0.42 **	0.03	0.30 *
Emotional	0.03	−0.15	−0.31 *	−0.14	0.05
Social	−0.27 *	−0.09	−0.28 *	−0.08	0.19
School	0.25 *	−0.13	−0.24	−0.11	−0.05
***PedsQL Total***	0.00	0.00	−0.41 ***	−0.07	0.17

*Note*. IIQ = Illness Identity Questionnaire; BRIEF-SR = Behavior Rating Inventory of Executive Function–Self-Report; SCMS = Self-Control and Self-Management; PedsQL = Pediatric Quality of Life Inventory; * *p* < 0.05, ** *p* < 0.01, *** *p* < 0.001.

**Table 3:** Summary of the model: predictors of diagnosis identity perception in stepwise multiple regressions.

Model	Predictor	B	*SE* B	β	*t*	*p*
1	Self-Monitoring (SCMS)	0.049	0.008	0.592	5.88	<0.001
		*F* (1,64) = 34.57, *p* < 0.001				
		*R* = 0.592, *R*^2^ = 0.351, *Adj. R*^2^ = 0.341				
2	Self-Monitoring (SCMS)	0.048	0.008	0.582	5.98	<0.001
	Social QoL (PedsQL)	0.007	0.003	0.231	2.37	0.021
		*F* (2,63) = 21.35, *p* < 0.001				
		*R* = 0.636, *R*^2^ = 0.404, *Adj. R*^2^ = 0.385				
3	Self-Monitoring (SCMS)	0.049	0.008	0.595	6.32	<0.001
	Social QoL (PedsQL)	0.007	0.003	0.226	2.40	0.019
	Self-Evaluation (SCMS)	0.029	0.012	0.221	2.35	0.022
		*F* (3,62) = 17.09, *p* < 0.001				
		*R* = 0.673, *R*^2^ = 0.453, *Adj. R*^2^ = 0.426				

**Table 4:** Summary of the model: Predicting EF, self-management, and QoL by IIQ dimensions in stepwise multiple regressions.

Outcome Variable	Predicator (IIQ Dimension)	B	*SE* B	β	*t*	*p*
Executive functions (BRIEF-SR GEC)	Engulfment	6.50	1.94	0.386	3.35	0.001
		*F* (1,64) = 11.20, *p* = 0.001				
		*R* = 0.386, *R*^2^ = 0.149, *Adj. R*^2^ = 0.136				
Self-management-Step 1	Enrichment	6.24	1.30	0.514	4.80	<0.001
		*F* (2,63) = 23.01, *p* < 0.001				
		*R* = 0.514, *R*^2^ = 0.264, *Adj. R*^2^ = 0.253				
Self-Management-Step 2	Enrichment	6.75	1.25	0.557	5.42	<0.001
	Engulfment	−4.05	1.43	−0.292	−2.84	0.006
		*F* (2,63) = 16.80, *p* < 0.001				
		*R* = 0.590, *R*^2^ = 0.348, *Adj. R*^2^ = 0.327				
Quality of life (PedsQL)	Engulfment	−6.52	1.83	−0.407	−3.56	0.001
		*F* (3,62) = 12.69, *p* < 0.001				
		*R* = 0.407, *R*^2^ = 0.165, *Adj. R*^2^ = 0.152				

## Data Availability

The data that support the findings of this study are available from the corresponding author upon reasonable request. Due to ethical considerations and the involvement of minors, the data are not publicly available to protect participant confidentiality.

## Abbreviations

The following abbreviations are used in this manuscript:

ADHD	Attention-deficit/hyperactivity disorder
BRI	Behavioral regulation index (BRIEF)
BRIEF	Behavior Rating Inventory of Executive Function
EF	Executive function
GEC	Global executive composite (BRIEF)
IIQ	Illness Identity Questionnaire
MI	Metacognition index (BRIEF)
PedsQL	Pediatric Quality of Life Inventory
QoL	Quality of life
SCMS	Self-Control and Self-Management Scale

## References

[1] American Psychiatric Association (2022). Diagnostic and Statistical Manual of Mental Disorders.

[2] Ayano G., Demelash S., Gizachew Y., Tsegay L., Alati R. (2023). The global prevalence of attention deficit hyperactivity disorder in children and adolescents: An umbrella review of meta-analyses. J. Affect. Disord..

[3] Fayyad J., Sampson N.A., Hwang I., Adamowski T., Aguilar-Gaxiola S., Al-Hamzawi A., Andrade L.H.S.G., Borges G., de Girolamo G., Florescu S. (2017). The descriptive epidemiology of DSM-IV adult ADHD in the World Health Organization World Mental Health Surveys. Atten. Deficit Hyperact. Disord..

[4] Kooij J., Bijlenga D., Salerno L., Jaeschke R., Bitter I., Balázs J., Thome J., Dom G., Kasper S., Filipe C.N. (2019). Updated European consensus statement on diagnosis and treatment of adult ADHD. Eur. Psychiatry.

[5] World Health Organization (2020). Basic Documents.

[6] Mitic M., Woodcock K.A., Amering M., Krammer I., Stiehl K.A.M., Zehetmayer S., Schrank B. (2021). Toward an integrated model of supportive peer relationships in early adolescence: A systematic review and exploratory meta-analysis. Front. Psychol..

[7] Gardner D., Gerdes A. (2015). A review of peer relationships and friendships in youth with ADHD. J. Atten. Disord..

[8] Grygiel P., Humenny G., Rębisz S., Bajcar E., Świtaj P. (2018). Peer rejection and perceived quality of relations with schoolmates among children with ADHD. J. Atten. Disord..

[9] Abecassis M., Isquith P.K., Roth R.M. (2017). Characteristics of ADHD in the emerging adult: An overview. Psychol. Inj. Law.

[10] Krauss A., Schellenberg C. (2022). ADHD symptoms and health-related quality of life of adolescents and young adults. Eur. J. Health Psychol..

[11] Adler L.A., Dirks B., Deas P., Raychaudhuri A., Dauphin M., Saylor K., Weisler R. (2013). Self-reported quality of life in adults with attention-deficit/hyperactivity disorder and executive function impairment treated with lisdexamfetamine dimesylate: A randomized, double-blind, multicenter, placebo-controlled, parallel-group study. BMC Psychiatry.

[12] Barkley R.A. (2015). Attention-Deficit Hyperactivity Disorder: A Handbook for Diagnosis and Treatment.

[13] Molitor S.J., Oddo L.E., Eadeh H.-M., Langberg J.M. (2019). Executive function deficits in adolescents with ADHD: Untangling possible sources of heterogeneity. J. Emot. Behav. Disord..

[14] Toglia J., Foster E.R. (2021). The Multi Context Approach to Cognitive Rehabilitation: A Metacognitive Strategy Intervention to Optimize Functional Cognition.

[15] Shimoni M., Engel-Yeger B., Tirosh E. (2012). Executive dysfunctions among boys with attention deficit hyperactivity disorder (ADHD): Performance-based test and parents report. Res. Dev. Disabil..

[16] Tonks J., Williams W.H., Slater A.M., Frampton I.J. (2017). Is damage to the pre-frontal cortex dormant until adolescence, or difficult to detect? Looking for keys that unlock executive functions in children in the wrong place. Med. Hypotheses.

[17] Blakemore S.J., Choudhury S. (2006). Development of the adolescent brain: Implications for executive function and social cognition. J. Child Psychol. Psychiatry.

[18] Al-Yagon M., Borenstein T. (2022). Adolescents’ executive functions: Links to inattention, hyperactivity-impulsivity, trait mindfulness, and academic performance. J. Adolesc..

[19] Barkley R.A. (2012). Executive Functions: What They Are, How They Work, and Why They Evolved.

[20] Weyandt L.L., Willis W.G., Swentosky A., Wilson K., Janusis G.M., Chung H.J., Turcotte K., Marshall S., Goldstein S., Naglieri J.A. (2014). A review of the use of executive function tasks in externalizing and internalizing disorders. Handbook of Executive Functioning.

[21] Munsell E.G.S., Orsmond G.I., Fulford D., Coster W.J. (2022). Self-management of daily life tasks in diploma-track youth with disabilities. Disabil. Rehabil..

[22] Lorig K.R., Holman H. (2003). Self-management education: History, definition, outcomes, and mechanisms. Ann. Behav. Med..

[23] Van de Velde D., De Zutter F., Satink T., Costa U., Janquart S., Senn D., De Vriendt P. (2019). Delineating the concept of self-management in chronic conditions: A concept analysis. BMJ Open.

[24] Araújo-Soares V., Hankonen N., Presseau J., Rodrigues A., Sniehotta F.F. (2019). Developing behavior change interventions for self-management in chronic illness: An integrative overview. Eur. Psychol..

[25] Audulv Å. (2013). The overtime development of chronic illness self-management patterns: A longitudinal qualitative study. BMC Public Health.

[26] Cronly J.A., Savage E. (2019). Developing agency in the transition to self-management of cystic fibrosis in young people. J. Adolesc..

[27] Lozano P., Houtrow A. (2018). Supporting self-management in children and adolescents with complex chronic conditions. Pediatrics.

[28] Sawyer S.M., Aroni R.A. (2005). Self-management in adolescents with chronic illness: What does it mean and how can it be achieved?. Med. J. Aust..

[29] Erikson E. (1968). Identity: Youth and Crisis.

[30] Dunn D.S., Burcaw S. (2013). Disability identity: Exploring narrative accounts of disability. Rehabil. Psychol..

[31] Raymaekers K., Prikken S., Vanhalst J., Moons P., Goossens E., Oris L., Weets I., Luyckx K. (2020). The social context and illness identity in youth with type 1 diabetes: A three-wave longitudinal study. J. Youth Adolesc..

[32] Luyckx K., Seiffge-Krenke I., Schwartz S.J., Goossens L., Weets I., Hendrieckx C., Groven C. (2008). Identity development, coping, and adjustment in emerging adults with a chronic illness: The sample case of type 1 diabetes. J. Adolesc. Health.

[33] Charmaz K. (1987). Struggling for a self: Identity levels of the chronically ill. Res. Sociol. Health Care.

[34] Oris L., Seiffge-Krenke I., Moons P., Goubert L., Rassart J., Luyckx K. (2016). Illness identity in adolescents and emerging adults with type 1 diabetes: Introducing the Illness Identity Questionnaire. Diabetes Res. Clinical Pract..

[35] Yanos P.T., Roe D., Lysaker P.H. (2010). The impact of illness identity on recovery from severe mental illness. Am. J. Psychiatr. Rehabil..

[36] Van Bulck L., Goossens E., Luyckx K., Oris L., Apers S., Moons P. (2018). Illness identity: A novel predictor for healthcare use in adults with congenital heart disease. J. Am. Heart Assoc..

[37] Luyckx K., Oris L., Raymaekers K., Rassart J., Moons P., Verdyck L., Mijnster T., Mark R.E. (2018). Illness identity in young adults with refractory epilepsy. Epilepsy Behav..

[38] Meyer S., Lamash L. (2021). Illness identity in adolescents with celiac disease. J. Pediatr. Gastroenterol. Nutr..

[39] Gray S.M., McMorris C.A., Mudry T.E., McCrimmon A.W. (2024). An exploration of diagnostic identity for autistic individuals: A systematic review of existing literature. Res. Autism Spectr. Disord..

[40] Lamash L., Sagie D., Selanikyo E., Meyer S., Gal E. (2024). Autism identity in young adults and the relationships with participation, quality of life, and well-being. Res. Autism Spectr. Disord..

[41] Lamash L., Meyer S. (2025). Confirmatory factor analysis of an identity questionnaire among autistic adolescents and young adults. Autism Dev. Lang. Impair..

[42] Abu Raya-Ghanayem N., Stern A., Lamash L. (2025). Diagnosis identity perception of adolescents with ADHD and its relationship to social participation and quality of life. Res. Dev. Disabil..

[43] Grimell J. (2025). Identity work among girls with ADHD: Struggling with Me and I, impression management and social camouflaging in school. Front. Psychol..

[44] Frick K., Skoglund C., Wästfelt E. (2024). ADHD and identity formation: Adolescents’ experiences from the healthcare system and peer relationships. BMC Psychol..

[45] Jones S., Hesse M. (2018). Adolescents with ADHD: Experiences of having an ADHD diagnosis and negotiations of self-image and identity. J. Atten. Disord..

[46] Oris L., Luyckx K., Rassart J., Goubert L., Goossens E., Apers S., Arat S., Vandenberghe J., Westhovens R., Moons P. (2018). Illness identity in adults with a chronic illness. J. Clin. Psychol. Med. Settings.

[47] Guy S.C., Isquith P.K., Gioia G.A. (2004). Behavior Rating Inventory of Executive Function–Self-Report Version (BRIEF-SR).

[48] Mezo P.G. (2009). The Self-Control and Self-Management Scale (SCMS): Development of an adaptive self-regulatory coping skills instrument. J. Psychopathol. Behav. Assess..

[49] Mezo P.G., Short M. (2012). Construct validity and confirmatory factor analysis of the Self-Control and Self-Management Scale. Can. J. Behav. Sci..

[50] Varni J.W., Seid M., Kurtin P.S. (2001). PedsQL™ 4.0: Reliability and validity of the Pediatric Quality of Life Inventory™ Version 4.0 Generic Core Scales in healthy and patient populations. Med. Care.

[51] Lamash L., Gutman Y., Meyer S., Gal E. (2024). Self & parent-reported autism identity perception among adolescents with ASD. Am. J. Occup. Ther..

[52] Mahdi S., Viljoen M., Massuti R., Selb M., Almodayfer O., Karande S., de Vries P.J., Rohde L., Bölte S. (2017). An international qualitative study of ability and disability in ADHD using the WHO-ICF framework. Eur. Child Adolesc. Psychiatry.

[53] Schippers L.M., Horstman L.I., Velde H.V.D., Pereira R.R., Zinkstok J., Mostert J.C., Greven C.U., Hoogman M. (2022). A qualitative and quantitative study of self-reported positive characteristics of individuals with ADHD. Front. Psychiatry.

[54] Kapp S.K., Gillespie-Lynch K., Sherman L.E., Hutman T. (2013). Deficit, difference, or both? Autism and neurodiversity. Dev. Psychol..

[55] Singh A. (2022). Neurodiversity in education: Celebrating differences and embracing strengths. Int. J. Res. Publ. Semin..

[56] Rasmussen L.A., Almvik A. (2024). “A bit lost”—Living with attention deficit hyperactivity disorder: A qualitative longitudinal study. BMC Psychol..

[57] Rasmussen L.A., Almvik A., Nortvedt P. (2018). Just being a kid, or an ADHD kid? A qualitative study of children’s experiences of attention deficit hyperactivity disorder. J. Atten. Disord..

[58] Barkley R.A. (1997). Behavioral inhibition sustained attention, and executive functions: Constructing a unifying theory of ADHD. Psychol. Bull..

[59] Diamond A. (2013). Executive functions. Ann. Rev. Psychol..

[60] Hirsch O., Chavanon M., Riechmann E., Christiansen H. (2018). Emotional dysregulation is a primary symptom in adult attention-deficit/hyperactivity disorder (ADHD). J. Affect. Disord..

[61] Hosseinzadeh H., Downie S., Shnaigat M. (2022). Effectiveness of health literacy- and patient activation-targeted interventions on chronic disease self-management outcomes in outpatient settings: A systematic review. Aust. J. Prim. Health.

[62] Kralik D., Koch T., Price K., Howard N. (2004). Chronic illness self-management: Taking action to create order. J. Clin. Nurs..

[63] Peters L., Brown E. (2022). The relationship between illness identity and the self-management of inflammatory bowel disease. Br. J. Health Psychol..

[64] Falkenberg C.A., Barbetta P.M. (2013). The effects of a self-monitoring package on homework completion and accuracy of students with disabilities in an inclusive general education classroom. J. Behav. Educ..

[65] Hoekstra T., Dam M., Klaassen G., Bos W.J.W., van der Boog P.J.M., Vogt L., van Jaarsveld B., van Dijk S., Navis G., Meuleman Y. (2025). Self-monitoring and self-efficacy in patients with chronic kidney disease during low-sodium diet self-management interventions: Secondary analysis of the ESMO and SUBLIME trials. Int. J. Behav. Med..

[66] Knödler L.L., Thomann P.A., Ebert M.P. (2020). The concept of illness identity in inflammatory bowel disease. J. Crohn’s Colitis.

[67] Karademas E.C., Karamvakalis N., Zarogiannos A. (2009). Life context and the experience of chronic illness: Is the stress of life associated with illness perceptions and coping?. Stress Health.

[68] Korfmacher A.-K., Hirsch O., Chavanon M.-L., Albrecht B., Christiansen H. (2022). Self-management training vs. neurofeedback interventions for attention deficit hyperactivity disorder: Results of a randomized controlled treatment study. Front. Psychiatry.

[69] Danielson M.L., Claussen A.H., Bitsko R.H., Katz S.M., Newsome K., Blumberg S.J., Ghandour R. (2024). ADHD prevalence among U.S. children and adolescents in 2022: Diagnosis, severity, co-occurring disorders, and treatment. J. Clin. Child Adolesc. Psychol..

[70] Mowlem F.D., Rosenqvist M.A., Martin J., Lichtenstein P., Asherson P., Larsson H. (2019). Sex differences in predicting ADHD clinical diagnosis and pharmacological treatment. Eur. Child Adolesc. Psychiatry.

